# Health information exchange policies of 11 diverse health systems and the associated impact on volume of exchange

**DOI:** 10.1093/jamia/ocw063

**Published:** 2016-06-14

**Authors:** N Lance Downing, Julia Adler-Milstein, Jonathan P Palma, Steven Lane, Matthew Eisenberg, Christopher Sharp, Northern California HIE Collaborative, Christopher A Longhurst

**Affiliations:** 1 Department of Medicine, Stanford University School of Medicine; 2 School of Information and School of Public Health, University of Michigan; 3 Department of Pediatrics, Stanford University School of Medicine; 4 Palo Alto Medical Foundation/Sutter Health, Palo Alto, CA,; 5 UC San Diego Health System

**Keywords:** electronic health record, EHR, HIE, high value care

## Abstract

**Background:** Provider organizations increasingly have the ability to exchange patient health information electronically. Organizational health information exchange (HIE) policy decisions can impact the extent to which external information is readily available to providers, but this relationship has not been well studied.

**Objective:** Our objective was to examine the relationship between electronic exchange of patient health information across organizations and organizational HIE policy decisions. We focused on 2 key decisions: whether to automatically search for information from other organizations and whether to require HIE-specific patient consent.

**Methods:** We conducted a retrospective time series analysis of the effect of automatic querying and the patient consent requirement on the monthly volume of clinical summaries exchanged. We could not assess degree of use or usefulness of summaries, organizational decision-making processes, or generalizability to other vendors.

**Results:** Between 2013 and 2015, clinical summary exchange volume increased by 1349% across 11 organizations. Nine of the 11 systems were set up to enable auto-querying, and auto-querying was associated with a significant increase in the monthly rate of exchange (*P* = .006 for change in trend). Seven of the 11 organizations did not require patient consent specifically for HIE, and these organizations experienced a greater increase in volume of exchange over time compared to organizations that required consent.

**Conclusions:** Automatic querying and limited consent requirements are organizational HIE policy decisions that impact the volume of exchange, and ultimately the information available to providers to support optimal care. Future efforts to ensure effective HIE may need to explicitly address these factors.

## INTRODUCTION

Health information exchange (HIE), the secure electronic sharing of health care–related data between organizations, remains an important unrealized source of return on the $40 billion public investment in health IT under the 2009 HITECH Act.^1–7^ For providers, HIE should result in better access to patient heath information from other settings to support clinical decision-making. Many policy efforts have targeted HIE expansion, but largely allowed different approaches to develop in the market, such as community-based exchange networks, enterprise-based exchange networks, and electronic health record (EHR) vendor-based platforms.

EHR vendor-based HIE networks are a relatively recent, and rapidly growing, type of HIE network that clinicians are increasingly likely to encounter in their organizations. While these networks feature some important limitations—in particular, the cost and complexity associated with connecting to other EHR vendors—they have gained traction because of key advantages. First, because exchange participants use a common software platform, they typically face fewer barriers to achieving interoperability. Second, some of these networks have established standard HIE governance models, which define “rules of the road” to address basic security standards, appropriate use of transmitted data, and explicit rules against information blocking.^8^

However, EHR vendor-based networks often leave other HIE configuration options and policy decisions to the participating provider organizations, and these decisions may have a significant impact on whether or not patient health information from other organizations is readily available to clinicians. The first important decision is whether to enable automatic search and retrieval of information. Because clinicians are often unaware when information about their patients exists in other provider organizations, such automatic searching can fill important information gaps. A downside to this approach is potential information overload for providers if the information is not presented in a concise and user-friendly fashion. A second important decision is whether to require that patients separately consent to having their information shared electronically with other provider organizations. Because such exchanges can be considered “treatment, payment, or operations” under HIPAA, explicit consent is not required by federal law and can be bundled with treatment consent. Minimizing patient consent reduces the burden on provider organizations engaging in HIE by eliminating a workflow step that can be time intensive, or even prohibitive in the case of automatic querying, when the patient may not be present at the time of the data request. Nonetheless, many provider organizations have chosen to take a more conservative approach and obtain explicit consent from patients every time their information is shared through an HIE network (or in certain cases, such as for any patient who has received mental health care). Little is known about these decisions made by individual provider organizations and the resulting impact on whether or not data follow patients throughout the care continuum.

In this study, we looked at data on organization-level HIE policy decisions and their impact on HIE volume from a diverse set of health care systems using the same EHR-based HIE platform. We focused on clinical summary exchange over a 2-year period (2013–2015) and asked the following research questions (1) What proportion of organizations chose to engage in automatic querying and what is the associated impact on volume of clinical summary exchange? (2) When automatic querying is enabled, what proportion of patient linkages are established automatically (representing information at another institution that the provider did not know to seek) vs manually (representing information the provider knew to seek)? (3) What proportion of organizations chose not to require patient consent for HIE and what is the associated impact on volume of clinical summary exchange? Understanding the impact of local organizational HIE policy decisions on the volume of exchange activity is important in ensuring that future efforts to promote HIE will consider these decisions. Without sufficient attention to these critical policies, technical interoperability may not translate into availability of needed health information for clinicians at the point of care.

## METHODS

### Setting

The Northern California HIE Collaborative comprises 12 institutions that use a common EHR vendor (Epic Systems, Verona, WI, USA) and its associated HIE platform (Care Everywhere). The collaborative formed over time through the efforts of one of the co-authors (S.L.) to connect regional organizations that had previously adopted Epic and turned on Care Everywhere, in order to share organizational policies and encourage organizations to lower barriers to exchange. The collaborative includes 3 university-affiliated academic systems (UC San Francisco, UC Davis, Stanford Health Care) and affiliated pediatric health systems (Stanford Children’s Health, Benioff Children’s Hospital Oakland), 2 large integrated delivery networks (Sutter Health and Kaiser Permanente Northern California), 2 safety net health care systems (Contra Costa County Health Services, Santa Clara Valley Medical Center), a network of community clinics linked by a common health information system (OCHIN), and 2 community health care systems (John Muir Health and Washington Hospital Healthcare System) (Table 1). Care Everywhere is a standards-based network providing peer-to-peer patient matching and query-based HIE between institutions. While the vendor maintains a directory of participating institutions, organizations connect directly with one another when attempting to match patients and when sharing health information on matched patients. For example, an organization seeking medical records for a patient seen in the emergency department would utilize the vendor-hosted organization directory to identify local organizations and then directly query those institutions for matching patient records. Since there is no universal patient number, organizations identify a patient match based on an algorithm incorporating name, address, date of birth, and other demographic data. If a match is found, then health information can be exchanged, subject to the sending institution’s patient consent requirement.

**Table 1:** Northern California HIE Collaborative: organizational characteristics and health information exchange policy decisions

	Integrated Health Care Network			Academic Medical Center					Safety Net Health System		Network of Community Clinics
	
	1	2	3	1a	1b	2a	2b	3	1	2	1
Org. Name	Sutter Health	John Muir Health	Washington Hospital Healthcare System	UCSF and affiliated Children’s Hospital San Francisco	UCSF- affiliated Children’s Hospital Oakland	Stanford Health Care	Stanford- affiliated Children’s Hospital	UC Davis Health System	Santa Clara Valley	Contra Costa County	OCHIN
Number of beds	4,334	817	353	649	191	613	311	619	574	176	N/A
Integrated health system	Yes	Yes	Yes	Yes	Yes	Yes	Yes	Yes	Yes	Yes	No
Emergency services	Yes	Yes	Yes	Yes	Yes	Yes	Yes	Yes	Yes	Yes	No
Pediatric services	Yes	Yes	Yes	Yes	Yes	Yes	Yes	Yes	Yes	Yes	Yes
HIE Organizational Policy Decisions											
Patient consent required	No	No	Yes	Yes	Yes	No	No	No	Yes^a^	No	No
Automatic querying enabled	Yes	Yes	Yes	Yes	No	Yes	Yes	No	Yes	Yes	Yes

^a^Consent required if a patient had a previous encounter in a confidential department (eg, mental health or substance abuse).

Participation in Care Everywhere requires agreeing to the vendor’s common governance standards, termed the “Rules of the Road.” In addition to basic security and privacy rules, this contract requires institutions to provide HIE access to other institutions regardless of competitive status, with the “goal of improving patient care by making additional patient information available to other providers.” Additionally, to resolve potential disputes, Epic Systems helps coordinate a member-run governing council.

However, within this vendor-sponsored HIE governance and technology platform, provider organizations have substantial latitude in local organizational policy decisions and the resulting configuration of how they engage in HIE through the network. Two key decisions are whether or not to enable auto-query and whether or not to require patient consent. Automatic querying functionality allows institutions to automatically look for patient matches at other institutions based on the patients they are treating, and to automatically download available information before an upcoming clinical encounter. For example, if a patient has an appointment at institution A, that health care system might be configured to query nearby health systems the night before and, if a match is found for that patient, to automatically download the patient’s data so that it is available for the following day’s appointment or surgery.

Patient consent (also called the patient authorization requirement) defines the requirement for every other institution that attempts to retrieve health information from the first institution. Options range from requiring patient authorization for every information transaction, to requiring authorization only when a patient has had an encounter in a confidential department (eg, mental health or substance abuse treatment), to no HIE-specific authorization requirement (ie, bundled as part of general treatment consent under HIPAA’s Treatment, Payment, or Operations). Of the organizations in the collaborative that required consent, patients had to sign a new point-of-care consent for every encounter in order to permit their information to be exchanged.

### Data

Data for this study came from 2 sources. Self-reported data was collected from chief medical information officers and other leaders at the participating provider organizations on their organizational policies, including auto-query (yes/no) and date of auto-query initiation, and approach to consent (none/for sensitive conditions/always). The second source of data was from 11 of the 12 collaborative organizations; 1 organization did not consent to share data for the purpose of the study. The EHR vendor reported standardized data for each institution’s monthly volume of patient linkages established, and clinical summaries retrieved, from every other organization within the collaborative (ie, at the dyad level). Linkage is measured as a successful patient match between 2 organizations, in response to either auto-query or manual query. Clinical summaries included information such as allergies, immunizations, medications, medical problems, medical and social history, advance directives, vital signs, and recent procedures and their results. Volume of information exchange has been noted as an important measurement in a recent report from the national HIE and Interoperability Measurement Community of Practice.^42^ While information exchanged does not guarantee clinical value, it is a necessary precursor. Lending further support to this measure, the Office of the National Coordinator for Health IT’s dashboard includes volume of query-based exchange as a national measure of the status of HIE in the United States.^43^ The data for our study covered a 2-year period from January 1, 2013, through February 28, 2015, and included linkages made and clinical summaries transferred across all clinical settings within each institution (outpatient, emergency department, inpatient, etc.). The Stanford University Institutional Review Board granted this study a non-human subjects exemption since these data contain no identifiable information about individual patients.

### Analytic approach

For descriptive purposes, we plotted the overall volume of exchange activity over time and depicted the dyadic exchange relationships using a circular visualization, with link thickness representing the ratio of data sent and retrieved between 2 institutions normalized across the institutions (circos.ca). For example, there may be a line with a thick end at organization A and a thin line at organization B, signifying that this partnership represents a large percentage of the summaries retrieved by organization A but a small percentage by organization B. Likewise, a line with thick ends on both sides signifies that both institutions retrieved a large percentage of their total summaries from each other.

To answer our first research question, regarding the relationship between auto-query and clinical summary exchange volume, we first used the *itsa* command in Stata to conduct an interrupted time series analysis for each organization that enabled auto-query to determine the change in level and trend of monthly volume of clinical summaries received after auto-query began.^9^^,^^41^ We limited the sample to the 6 provider organizations with at least 3 months of data post auto-query initiation. We then used the time-series variables created by *itsa* to assess the relationship across all organizations with a general estimating equation model, which accounted for the serial correlation within organizations over time.

To answer our second research question, regarding the proportion of patient links established through auto-query vs manually, we first limited our data to exchange activity during January and February 2015. This captured a period after all provider organizations had started sharing summaries through Care Everywhere and also after all organizations that chose to turn on auto-query had done so. Then, for each organization, we summed the number of patient linkages established through auto-query and those established manually during the 2-month period.

In Care Everywhere, the patient-matching strategy is the same with manual or auto-query; any patient that can be linked across 2 organizations via manual query can also be linked via auto-query. If an organization has auto-query enabled, there is an attempt to establish a patient link for each visit, and if there is information available, the link is established. If an organization does not have auto-query enabled, a user must take the time to seek out information before or during the visit. Then, if a patient link is found at another organization, a link is established. After a patient link between 2 organizations is established, the standard workflow is to transfer a patient-level CCD, the most recent encounter CCD, and a list of additional encounters for which a CCD is available for transfer. Once the link is established, whether done manually or by auto-query, it persists and is available to all future users who open the patient record. However, for institutions requiring point-of-care patient authorization, providers must obtain patient consent to review external records for each new encounter; the authorization does not persist between visits and must be obtained for each encounter, whether with the same pair of organizations or a new pair (Supplemental Figure 1).

To answer our third research question, regarding the relationship between patient consent and clinical summary exchange volume, we examined the monthly volume of clinical summaries sent by each organization. Because consent policies did not vary within organizations over time, we used a linear regression model with 3 independent variables: time (a monthly counter starting at 1 in the first month of Care Everywhere participation for each organization), no_consent (a dichotomous variable coded 1 for organizations that did not require specific consent for HIE and 0 for those that did, and an interaction term between time and consent to assess whether the relationship between time and volume of clinical summaries sent varied by approach to consent. We used clustered standard errors by provider organization. We also ran a robustness test in which the model was limited to the post auto-query period for organizations that initiated auto-query.

## RESULTS

### Organizational HIE policy decisions

Nine of the 11 provider organizations (82%) enabled automatic querying during the study period. UCSF-affiliated Children’s Hospital Oakland and UC Davis Health System did not. Consent decisions were more varied. Four of the 11 provider organizations (36%) required point-of-care patient authorization, 1 (9%) required authorization if a patient had a prior encounter in a confidential department (eg, mental health), and 6 organizations (54%) did not require any HIE-specific authorization (Table 1).

### Total volume of exchange

**Figure 1:**
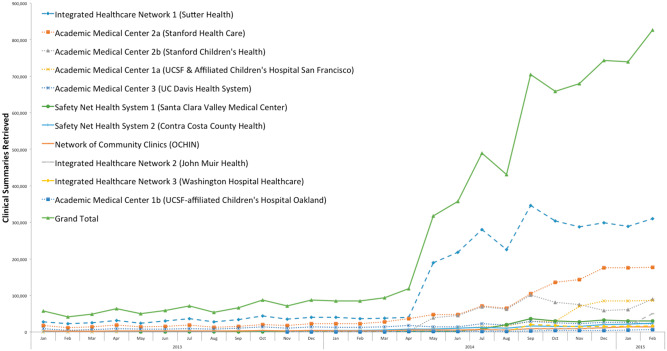
Overall trends in clinical summaries exchange volume: 2013–2015 *Note*: Volume of clinical summaries retrieved by each organization

A total of 6 909 416 clinical summaries were retrieved by participating organizations within the regional HIE network over the time frame of the study. In January 2013, 6 organizations were participating and retrieved 57 000 clinical summaries. By February 2015, all 11 organizations were participating and retrieved 826 000 clinical summaries, representing a 1349% increase in exchange volume (Figure 1).

**Figure 2:**
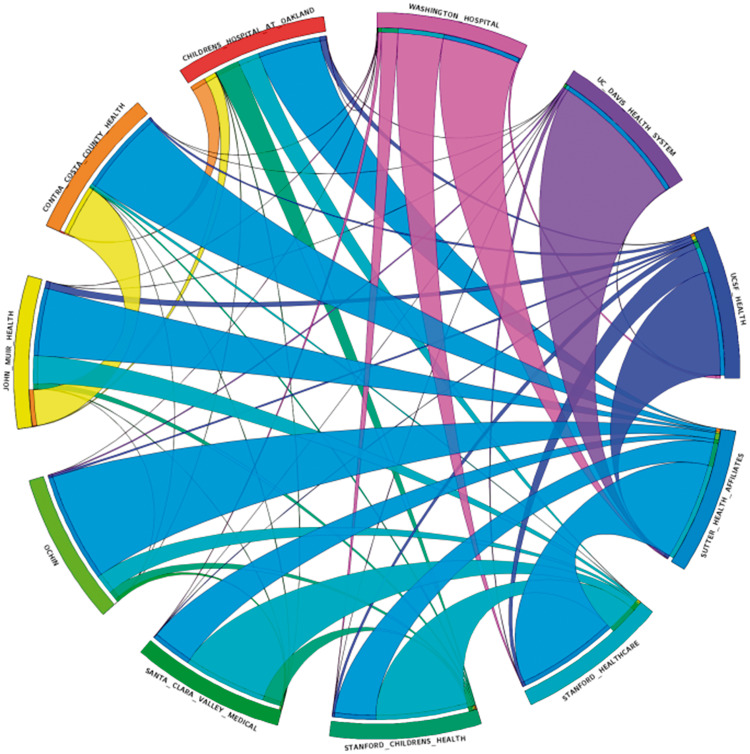
Clinical summaries sent and received between organizations. Link thickness represents ratio of information sent and retrieved between 2 institutions normalized across the institutions (circos.ca).

Examining exchange volume relationships dyadically reveals substantial asymmetry between exchange patterns (Figure 2). Each organization participated to some degree in information exchange with every other organization in the collaborative, except that the UC Davis Health System did not retrieve any information from Washington Hospital (likely due to geographic referral patterns). Additionally, there were some pairs with relatively equal bidirectional sharing with each other (eg, Sutter Health and Stanford Health Care) and other pairs in which the exchange was largely one-sided (eg, UC Davis Health System and Sutter Health).

### Impact of auto-query

**Figure 3:**
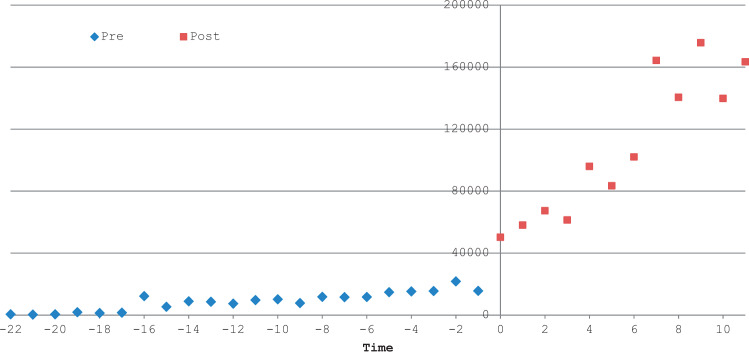
Impact of auto-query on volume of clinical summaries received *Note:* This exhibit reports results from an interrupted time series analysis on volume of clinical summaries received from other collaborative organizations; data come from the 6 organizations with at least 4 months of data post auto-query go-live.

The initiation of auto-query resulted in a significant increase in the monthly volume of clinical summaries received (Figure 3). In the pre auto-query period, exchange volume increased by 635 summaries per month. In the post auto-query period, exchange volume increased by 9929 summaries per month (P = .006 for change in trend, Table 2). We did not observe a significant change in level (coefficient = 38 435; P = .239, Table 2). Table 2 also reports interrupted time series analysis results for each provider organization individually.

**Table 2:** Interrupted time series models: impact of auto-query on volume of clinical summaries retrieved

	Estimated monthly change in volume of clinical summaries retrieved: pre *vs* post auto-query					Estimated change in volume of clinical summaries retrieved occurring immediately following the start of auto-query	

	Pre		Post		*P*-value (Pre vs Post)	Intercept change (95% CI)	*P*-value
	Months	Monthly Trend (95% CI)	Months	Monthly Trend (95% CI)			
Integrated Network 1	16	1108 (86, 2131)	10	6322 (−3020, 15 700)	.262	191 982 (137 783, 246 180)	<.001
AMC 1a	22	1063 (441, 1685)	4	1124 (−2550, 4799)	.973	55 805 (42 815, 68 795)	<.001
AMC 2a	14	524 (−82, 1131)	12	13 600 (10 600, 16 500)	<.001	1643 (−15 412, 18 700)	.843
Safety Net 1	19	292 (−43, 629)	7	81 (−2560, 2720)	.869	25 163 (13 352, 36 975)	<.001
Safety Net 2	22	728 (525, 930)	4	2390 (2190, 2590)	<.001	1325 (−1360, 4012)	.317
Community Clinics 1	19	193 (105, 282)	7	1030 (570, 1490)	.001	1182 (−916, 3280)	.255
Combined	22	635 (−105, 1376)	12	9929 (4443, 15 415)	.006	38 435 (−32 263, 1091)	.239

### Automatic vs manual patient links

**Figure 4:**
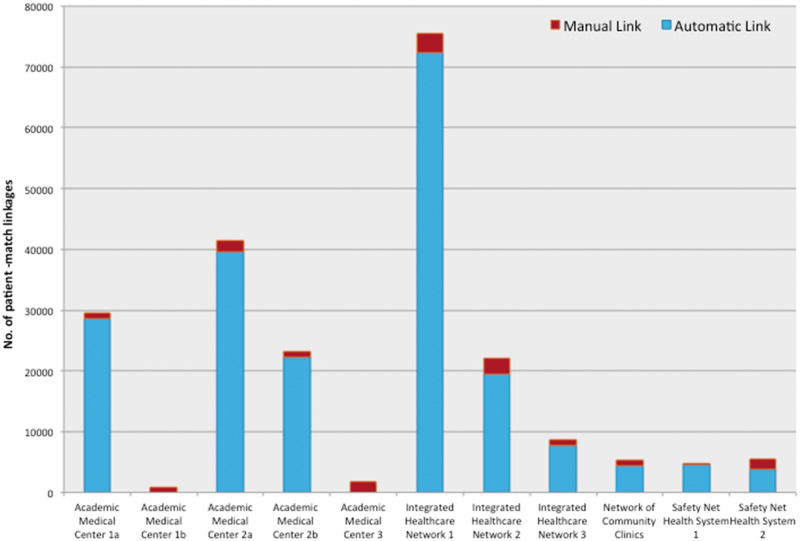
Total patient-match linkages by auto-query vs manual query

Patient links established by auto-query vastly outnumbered those established manually (Figure 4). Across the 9 provider organizations that enabled auto-query, the ratio of automatic to manual links ranged from 2:1 to 31:1.

### Impact of patient consent

**Figure 5:**
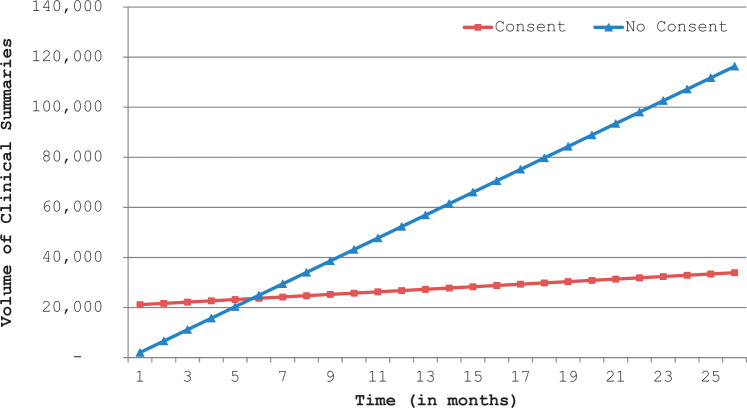
Impact of patient consent requirements on volume of clinical summaries sent Note: This exhibit reports results from a longitudinal model that assessed the volume of clinical summaries sent to other collaborative organizations based on whether or not the organization sending the clinical summaries required patient consent prior to the clinical summary being sent. All 11 organizations are included in this model.

Provider organizations that required consent experienced a marginally statistically significant increase in clinical summary exchange volume of 510 summaries per month (P = .066, Table 3). Provider organizations that did not require consent experienced a significantly greater increase of 4571 summaries per month (P = 0.042, Table 3). While no consent organizations started at a lower exchange volume overall, after 6 months they surpassed the consent organizations (Figure 5). Results were similar when we limited the data to the post auto-query period (Table 3).

**Table 3:** Longitudinal models: impact of patient consent requirements on volume of clinical summaries sent

	Model 1:		Model 2:	
	All months for all organizations (*N* = 11)		All months for no auto-query organizations (*N* = 2) only months post auto-query for auto-query organizations (*N* = 9)	
	Monthly trend (95% CI)	*P*-value	Monthly trend (95% CI)	*P*-value
Time	510 (−41, 1062)	.066	609 (−207, 1427)	.127
No consent	−19 114 (−34 786, −3443)	.022	−71 329 (−205 308, 62 650)	.263
Time*no consent	4571 (200, 8941)	.042	7655 (−14 954, 3700)	.067

## DISCUSSION

To our knowledge, this is the first large-scale empirical study to examine local HIE organizational policy decisions in a diverse group of health care systems and assess their impact on the volume of information exchange. While exchange volume does not guarantee clinical value of exchanged information, it is a mandatory prerequisite, and the need for studies that detail these types of implementation policies and configuration decisions has been noted as an important gap in the literature.^10–13^ This study focuses on 2 important policy decisions, auto-query and patient consent, and reveals that both are influential in ensuring that patient data is exchanged across separate health systems and disparate providers, a key first step in obtaining value from health information exchange. Specifically, both enabling automated patient record querying and information retrieval and minimizing patient consent processes appeared to substantially increase exchange volume. Perhaps most critical is our finding that auto-query identifies a large volume of information that was not sought out manually, suggesting that providers either did not know about this information, did not have the time to retrieve it, or did not consider it to be clinically relevant. These findings highlight that local organizational policy decisions have a significant impact on the extent to which information is available to clinicians to support decision-making.^11^^,^^14–24^ While not all of the data will be valuable to providers, making it easily accessible, as well as simply making the provider aware that care has been received elsewhere, is an important part of the HIE value proposition. Future emphasis on these nontechnology factors is important in ensuring that HIE capabilities translate into more complete information and improved care.

As with other aspects of health information technology, usability and workflow are critical to the successful adoption of HIE. We find a significant increase in the rate of clinical summary exchange after enabling automatic querying among applicable organizations. Furthermore, for organizations performing auto-query, the vast majority of patient linkages are established this way rather than by manual querying. This indicates that there may be significant numbers of patients with records elsewhere that are not identified when relying on manual querying alone. Automatic querying is an example of a technologically feasible functionality that improves workflow for providers by decreasing the burden of seeking outside patient records. This is important for busy clinicians who may not have the time to actively seek outside records if they are not readily available at the point of care. This and other functionalities that decrease workflow barriers to health information exchange should be prioritized in HIE platforms.^25–30^

Previous studies have identified the patient consent process as critical to HIE.^25^^,^^31–33^ In the Northern California HIE Collaborative, we observed wide variation in patient authorization requirements despite common state and local regulations. Among institutions with no HIE-specific patient consent requirement, some bundle HIE consent as part of the consent for treatment while still allowing an opt-out option. The institutions with no HIE-specific consent requirement demonstrated higher rates of HIE growth compared to those with explicit patient consent requirements. This is likely because streamlining or bundling the authorization process, while maintaining privacy and respecting patient preferences, lowers barriers to information exchange and represents a low-cost strategy to increase health information sharing. Going forward, it will be important to understand in more depth how organizations decide on their consent policy and how they implement their chosen approach, topics beyond the scope of the current study. We are encouraged, however, by the fact that, since data collection for this study occurred, several of the collaborative institutions bundled consent and enabled auto-query. Anecdotally, organizational leaders stated that having regional partners that set the precedent of streamlining HIE consent and enabling the auto-query process paved the way for them to follow suit, largely by assuaging concerns from compliance departments.

While our results do not directly speak to the reasons why organizations make different HIE policy decisions, there is some evidence that the need to gain patient trust and the legal ambiguity around patient consent when engaging in HIE may influence health care organizations to adopt more conservative consent policies.^34^ Similarly, organizations may not enable auto-query due to privacy, risk management, or competitive concerns. To the extent that these reasons are driving organizational HIE policy decisions, policy-makers may need to take targeted action: by explicitly specifying that HIE falls within HIPAA’s Treatment, Payment, and Operations, and by encouraging minimal consent processes, policy-makers could significantly increase HIE at minimal cost while preserving patient privacy. Strategies to incentivize automatic querying and improve usability could also spur large increases in exchange, making patient data that providers were previously unaware of available at the point of care.

Our findings not only demonstrate the variation in exchange volume from local organizational policy decisions, but also suggest a broader factor that enables increased exchange despite competitive concerns that can inhibit provider organizations’ participation in HIE. Specifically, Epic Systems employs an “all or nothing” approach: any provider organization that participates in its exchange network must wholly agree to the “Rules of the Road” that define appropriate use of transmitted data as well as other governance issues (though they are governed by representatives from client sites and therefore could be altered based on client feedback). This avoids the need for participating provider organizations to negotiate terms with every other organization in the network, which can be particularly challenging when these organizations consider each other competitors. In light of a recent report to Congress on health information blocking (the intentional practice of interfering with health information exchange, often for competitive reasons), efforts to promote simple, standardized terms to which all participating organizations agree may help to increase provider organization engagement in HIE networks, particularly in competitive health care markets.^8^^,^^35–39^

### Limitations

Since census and visit data for each institution were not accessible, exchange volume could not be normalized to account for the volume of patient care. However, because we have 2 years of data, we are still able to draw meaningful conclusions on the relationship between local HIE policy decisions and volume of clinical summary exchange overtime. Additionally, we are unable to determine which clinical summaries were actually utilized for patient care and therefore offered information of clinical value. However, access to data from other institutions is a necessary condition for clinician use, and ultimately improved care outcomes. We were also unable to observe the HIE policy decision-making processes of provider organizations or how they implemented their approach to auto-query or consent. Finally, this study included only institutions using a single vendor-based HIE platform. Therefore our findings may not be generalizable to other approaches to HIE, because Epic’s Care Everywhere is somewhat unique in terms of its approach to technical interoperability and standards governance. However, this is what allows us to focus on the important area of organizational policy decisions in which little prior work exists.

In summary, this study represents the first insights into organizational HIE policy decisions and the resulting impact on volume of exchange in the context of the growing trend of EHR vendor-based HIE platforms. In this approach to HIE, a nonnegotiable set of rules was accepted by competing organizations to allow exchange to take place, and simple, bundled patient consent requirements as well as automated patient record matching and retrieval significantly increased the extent to which information from other settings was available to providers. Future efforts to ensure that HIE capabilities translate into availability of patient information at the point of care, and ultimately improved outcomes, will likely need to broaden their focus to include these important factors.^4^^,^^11^^,^^23^^,^^40^

## Supplementary Material

Supplementary Data
