# Supplementary material for: Health information exchange policies of 11 diverse health systems and the associated impact on volume of exchange
Source: J Am Med Inform Assoc. 2016 Jun 14;24(1):113–22. doi: 10.1093/jamia/ocw063 (PMC7654085; doi:10.1093/jamia/ocw063)
Supplement: Supplementary Data [file ocw063_supp.docx]

Supplemental Figure 1: Workflow diagram for auto-query and manual query with and without explicit consent process.

Supplemental Table 1: Interrupted Time Series Models: Impact of Auto-Query on Volume of Clinical Summaries Retrieved

|  | **Change in Trend** | | | | | **Change in Level** | |
| --- | --- | --- | --- | --- | --- | --- | --- |
|  | Pre | | Post | | P-Value  (Pre vs. Post) | Intercept Change  [95% CI] | P-Value |
|  | Months | Monthly Trend  [95% CI] | Months | Monthly Trend  [95% CI] |  |  |  |
| Int Network 1 | 16 | 1,108  [86, 2,131] | 10 | 6,322  [-3,020, 15,700] | 0.262 | 191,982  [137,783, 246,180] | <0.001 |
| AMC 1a | 22 | 1,063  [441, 1,685] | 4 | 1,124  [-2,550, 4,799] | 0.973 | 55,805  [42,815, 68,795] | <0.001 |
| AMC 2a | 14 | 524  [-82, 1,131] | 12 | 13,600  [10,600, 16,500] | <0.001 | 1,643  [-15,412, 18,700] | 0.843 |
| Saf Net 1 | 19 | 292  [-43, 629] | 7 | 81  [-2,560, 2,720] | 0.869 | 25,163  [13,352, 36,975] | <0.001 |
| Saf Net 2 | 22 | 728  [525, 930] | 4 | 2,390  [2,190, 2,590] | <0.001 | 1,325  [-1,360, 4,012] | 0.317 |
| Comm Clinics 1 | 19 | 193  [105, 282] | 7 | 1,030  [570, 1,490] | 0.001 | 1,182  [-916, 3,280] | 0.255 |
| **COMBINED** | **22** | **635**  **[-105, 1,376]** | **12** | **9,929**  **[4,443, 15,415]** | **0.006** | **38,435**  **[-32,263, 1,091]** | **0.239** |

Supplemental Table 2: Longitudinal Models: Impact of Patient Consent Requirements on Volume of Clinical Summaries Sent

|  | **Model 1:**  All Months for All Organizations (N=11) | | **Model 2:**  All Months for No Auto Query Organizations (N=2)  Only Months Post Auto-Query for Auto-Query Organizations (N=9) | |
| --- | --- | --- | --- | --- |
|  | Monthly Trend  [95% CI] | P-Value | Monthly Trend  [95% CI] | P-Value |
| Time | 510  [-41, 1,062] | 0.066 | 609  [-207, 1,427] | 0.127 |
| No Consent | -19,114  [-34,786, -3,443] | 0.022 | -71,329  [-205,308, 62,650] | 0.263 |
| Time*No Consent | 4,571  [200, 8,941] | 0.042 | 7,655  [-14,954, 3,700] | 0.067 |
